# Spatial variability and temporal changes in the heavy metal content of soils with a deep furrow-and-ridge microrelief formed by an afforestation plowing

**DOI:** 10.1007/s10661-012-2931-3

**Published:** 2012-10-20

**Authors:** Cezary Kabala, Oskar Bojko, Agnieszka Medynska, Agnieszka Szczepaniak

**Affiliations:** Institute of Soil Science and Environmental Protection, Wrocław University of Environmental and Life Sciences, Grunwaldzka 53, 50-375 Wrocław, Poland

**Keywords:** Soil monitoring, Sampling strategy, Soil variability, Copper, Zinc

## Abstract

An appropriate sampling method that provides for the representation of the collected material and the reliability of results plays a crucial role in environmental monitoring. This is especially important in soil quality investigations on sites with a differentiated surface microrelief, as in the case of afforested post-arable soils that have a specific, deep furrow-and-ridge microrelief. The present research was carried out on three sites afforested with pine (4-, 8-, and 15-year-old stands) located near a large tailings pond collecting the wastes from copper ore enrichment. Soils were sampled at depths of 0–10 and 0–30 cm, separately in the furrows and ridges. The “wide-furrow plow” contributed to the spatial variation in soil properties, including higher pH, organic carbon, and Cu content in soils of the ridges. The difference in Cu content in the ridges and furrows initially reached 300 %, and decreased with the decline of the furrow-and-ridge microrelief to 60 % at 15 years after the plowing. Observed rate of the furrow shallowing allows for an estimation of the time necessary for the complete disappearance of the furrow-and-ridge microrelief and associated variability in soil properties to at least 30–40 years after the plowing. Afforestation plowing had little impact on the Zn variability which was not influenced by the emissions from the tailings pond. Soil sampling in contaminated sites with furrow-and-ridge microrelief must collect equal quantities of soil samples from both furrows and ridges to allow a reliable estimation of the mean trace elements’ concentration.

## Introduction

**Fig. 1:**
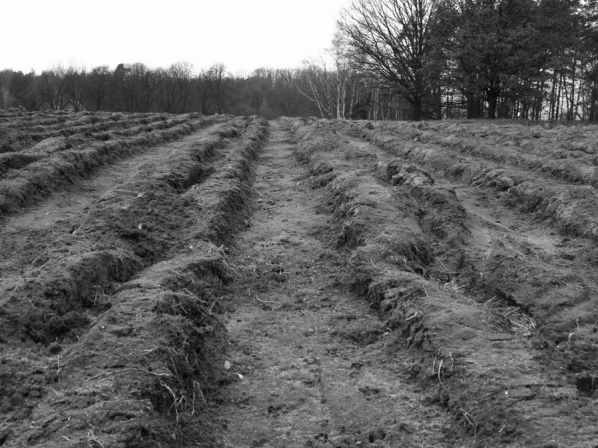
“Fresh” furrow-and-ridge microrelief created recently by an afforestation plowing (“wide-furrow plow”) in the surrounding of the study sites (an area not involved in this study)

Monitoring investigations, especially long-term measurement series, are subject to a number of threats to the reliability of their results: there are potential errors during sampling or preparation of the representative sample in the field, errors during sample preparation for analysis, and errors associated with laboratory analysis and data computing (Kempthorne and Allmaras 1985). The sum of their effects leads to an excessive seasonal or annual variability of observations within a long-term series and impedes the identification of a dominant time trend (Kabala et al. 2008a; Lark et al. 2006). However, the laboratory procedures have been increasingly improved due to constant modernization of analytical techniques and instrumentation, and validation and accreditation of laboratories, which finally increases the precision and accuracy of laboratory analyses (Swyngedouw and Lessard 2008). Therefore, the methodology of field work may have the greatest impact on the representativeness of the analytical material, and the errors associated with sample collection may diminish the comparability and reproducibility of monitoring data (Karczewska et al. 2010, Pennock et al. 2008). These problems affect all the observed and sampled components of the environment, including soils, especially their surface layer, whose spatial variability is influenced by geological, geomorphological, climatic, biotic, and anthropogenic factors (Souza et al. 2006, Szopka et al. 2010, 2011). For this reason, a number of different soil sampling strategies which aim to overcome or properly reflect soil variability are applied in soil quality monitoring. Soil variability—at a local scale—is relatively less significant on arable fields, where the soil is regularly averaged by agrotechnical treatment (Arrouays et al. 2000; Buccolieri et al. 2010; Wopereis et al. 1988), and relatively more significant on extensively cultivated or semi-natural areas influenced by soil fauna activity, and the most significant—under semi-natural forests, where each individual old tree may create a specific soil variability in its immediate surroundings (Belanger and Van Rees 2008; Bradford et al. 2010). Particularly large natural variations of soil properties, associated with fluctuations in the local relief and microrelief, have been reported from the mountains (Karczewska et al. 2006) and wetlands, especially on recently formed floodplains (Gallardo 2003). The vast majority of soil sampling methods assume a random spatial distribution of soil variability, particularly at the micro- and local scale (Guo-Shun et al. 2008; Lin et al. 2005; Martin et al. 2006). These strategies may fail if the spatial variability is at least partly systematic or ordered, often due to anthropogenic impact (Cannon and Reid 1993), as in the case of areas plowed in wide furrow. Such problems may occur in contaminated soils on newly afforested former farmlands, where tree seedlings were planted in deep furrows. This type of plowing is widespread in Polish forestry due to several advantages for seedling growth during the first 2–3 years after planting (Fig. 1). The deep furrows provide better availability of water for the plants, create a protective microclimate, and reduce competition from weeds (Gil 2006). However, plowing the furrows on contaminated sites causes a segregation of the contaminated topsoil onto the ridges between which, in the furrows, less contaminated subsoil is revealed. Such segregation creates differences in soil properties, including texture, pH, organic matter, and trace element content in soil on the ridges and in the furrows, especially on sandy soils. Soil sampling programs that ignore this kind of space variability lead to an extremely large variation in the element content during long-term observations (Kabala et al. 2008b). Over time, ridges are eroded leading to the disappearance of the specific furrow-ridge microrelief of the soil surface that should result in a successive diminishing of the trace metal variability in the monitoring site. However, there are no data in the scientific literature on the period or rate of a disappearance of the furrow-and-ridge microrelief and the associated strip-type variability of soil properties under the forest.

**Fig. 2:**
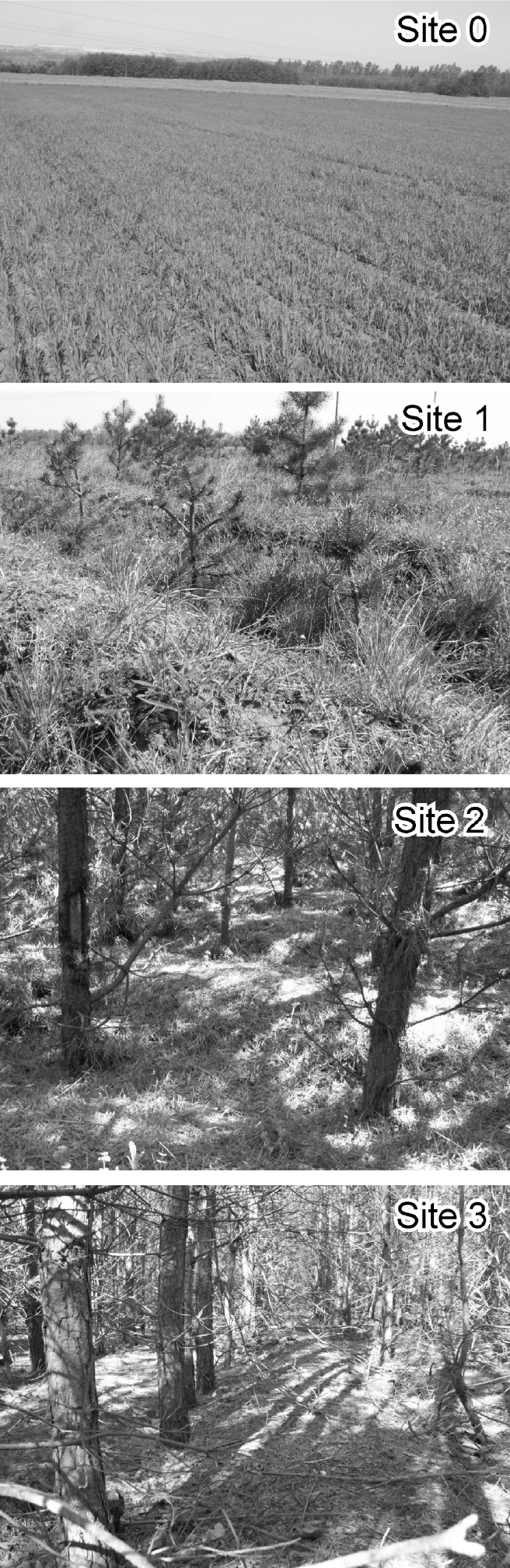
Reference arable land with flat microrelief (site 0) and the “furrow-and-ridge microrelief” on sites afforested 4, 8, and 15 years previously (sites 1, 2, and 3, respectively)

The aims of this work were to analyze the initial differentiation of soil properties and trace element content in the surface layer in a 15-year series of afforested post-arable soils with furrow-ridge microrelief of the surface and to assess the rate of a microrelief decline. The overall objective of the study was to develop a correct methodology for soil monitoring in the area surrounding industrial and mining installations, where a part of the agricultural land was afforested within a so-called sanitary zone protecting human settlements from dust emission.

## Materials and methods

### Source of contamination

The study was conducted in the vicinity of a large tailings pond that collects the wastes from copper ore enrichment (by the flotation method) located in Rudna (Lubin county, southwest Poland). The operation of the tailings pond began in 1977, and up to now, it has collected over 500 million m^3^ of waste on the area of over 1,670 ha (Kabala et al. 2008b). The tailings pond in Rudna is the largest in Poland and one of the largest in Europe.

The tailings are neutral or slightly alkaline (pH 7.5–7.8) and consist mainly of quartz, silicates, and carbonates, mixed in different proportions—depending on the local lithology of the ore (copper mineralization occurs in Permian sandstones, dolomites, and shales). Tailings have small amounts of minerals containing copper, lead and arsenic, and also trace amounts of zinc, nickel, vanadium, mercury, cobalt and a number of other elements (Luszczykiewicz 2000).

The dominant mineral fraction of the tailings is silt (particle size range between 0.002 and 0.05 mm); its share varies between 40 and 95 % of the sediment (by weight). Particle size distribution (texture of tailings) differs significantly from the landfill center to its border (“crown”) due to particle segregation during sedimentation in the pond (Kijewski and Downorowicz 1987). The landfill is raised above the surrounding area (up to 62 m in its eastern part) so its surface is highly exposed to the strong west winds. Emerged tailings rapidly dry up and form broad “beaches” in the peripheral zone of the pond. Dry, silt-sized particles are susceptible to eolian deflation; thus, emissions from the tailings pond are a serious environmental problem (Luszczykiewicz 2000). Alkaline dust in the air directly affects human and animal health, deteriorates the quality of the agricultural products and animal fodder, and also contaminates soil with heavy metals. Several attempts have been undertaken to prevent the dust blowing out of the landfill, such as covering the dry tailings with a thin bitumen coating or making an artificial fog (“water curtain”) in order to moisten dust particles suspended in the air and quicken their sedimentation.

Observations of the air, ground water, and soil quality started directly after construction of the tailings pond (Kabala et al. 2008b). Most exposed to contamination is the area located east of the landfill (due to the prevailing western winds), close to the village of Rudna, and also in this region are concentrated the air and soil monitoring sites.

### Sampling sites and sampling procedure

The study area was located on the eastern foreland of the tailing pond, about 400 m from the landfill. Soil sampling sites were established on three areas planted with pine (*Pinus sylvestris* L.) with a small admixture of birch (*Betula pendula* Roth.), with the control plot (site 0) on an arable field. The pine stands were 4, 8, and 15 years old (on sites 1, 2, and 3, respectively), meaning the period following a “wide-furrow plow” and the period of furrow-and-ridge microrelief decline (Table 1). Soils were classified according to WRB (IUSS 2006) as Brunic Arenosols (Dystric) or according to Soil Taxonomy (USDA 2010) as Typic Quartzipsamments. Before tree planting, the wide furrows were plowed to a depth of ca. 18 cm creating a furrow-and-ridge microrelief with an average (initial) depth of 32–35 cm at each site. Such a “fresh” and deep microrelief may be observed elsewhere in the surroundings, where new forest plantations are created on the post-arable fields (Fig. 1). The depth of the microrelief was measured at least 15 points located in the central part of three adjacent furrows. The surface of the furrows and ridges was cleaned of litter and vegetation at the measurement site, and a 1-m-long wooden beam resting on two adjacent ridges indicated the height of microrelief. Similarly, at each site, 15 primary soil samples were collected in three adjacent furrows and ridges, respectively. Soil samples, both on the ridges and in the furrows, were taken at 1-m intervals and at two depths separately: 0–30 cm in accordance with the requirements of Polish law (the standard of soil quality, act No Dz.U.2002.165.1359) and 0–10 cm, e.g., from the superficial layer, where the most dynamic changes were expected. All soil samples were collected using a stainless steel rill sampler with a diameter of 35 mm. After drying, soil samples were ground and quartered to obtain a representative sample for analysis.

**Table 1:** Basic characteristics of the sites

Site	Age of the stand (years)	Microrelief depth (cm)	Stand composition	Soil unit according to WRB
0	–	–	Arable field	Brunic Arenosol (Dystric)
1	4	28 ± 3a	Pine (95 %), Birch (5 %)	Brunic Arenosol (Dystric)
2	8	20 ± 3b	Pine (95 %), Birch (5 %)	Brunic Arenosol (Dystric)
3	15	15 ± 2c	Pine (100 %)	Brunic Arenosol (Dystric)

Homogeneous groups of means calculated by Duncan’s multiple range test (significant at *p* < 0.05). Different lowercase letters indicate significant differences between the mean microrelief depths (a lack of homogenous groups)

### Analytical methods

The following soil properties were analyzed using standard laboratory procedures (Van Reeuwijk 2002): particle size distribution—by sieving (sand fraction) and by hydrometer method (silt and clay fractions) following pretreatments to remove organic matter, and chemical dispersion with sodium hexametaphosphorate; pH in distilled water and in 1 M KCl solution (soil to solution ratio of 1:2.5)—potentiometrically; and organic carbon content by the dry combustion method using an automatic CS-mat 5500 apparatus (Ströhlein, Germany). Total concentrations of copper and zinc were measured using atomic absorption spectrophotometry (Philips PU9100) in extracts obtained by sample digestion with aqua regia mixture (HCl/HNO_3_ as 3:1) in a microwave oven (ISO 11466). The quality of determinations has been monitored using soil reference materials (SRM 2709, SRM 2711, RTH 912, and RTH 953) with the certified total concentration (“aqua regia extractable”) of trace elements being analyzed. Copper in soils surrounding the tailings pond is considered to be an indicator of contamination levels, and zinc generally occurs in natural amounts and is considered to be close to the geochemical background (Kabala et al. 2008b; Medynska et al. 2009). In this work, zinc was included as a reference metal for copper. Basic statistical calculations (mean and standard deviation) and the proof of the statistical significance of differences (Duncan test) were performed using the Statistica 9 package.

## Results and discussion

The depth of furrow-and-ridge microrelief, measured at the mineral soil surface (without forest litter), averaged 28 cm 4 years after the plow and tree planting (Table 1 and Fig. 2, site 1). In the subsequent years, a clear shallowing of the furrows has been observed: the average depth of furrows has reduced to 20 cm after 8 years (site 2) and 15 cm after 15 years following plowing (site 3). The depth of the furrows under a 15-year-old stand is apparently even smaller due to accumulation of forest litter that is much larger in the furrows than on the ridges. The rate of microrelief decay was higher during the first 10 years (when it reached 20 mm year^−1^), e.g., before the formation of a dense cover of forest litter. Thereafter, the decay rate decreased to less than 10 mm year^−1^ (on average 7.1 mm year^−1^ in the period 8–15 years after plowing). Ad hoc measurements made in two other pine stands (located at larger distances from the landfill, and not involved in this study) showed that the furrow-and-ridge microrelief is clearly visible three decades after the plowing, and even under 40-year-old pine stands has still not disappeared completely, forming a-few-centimeter-thick (8 cm on average) soil surface inequality.

All soils in the study sites had similar textures—in the sandy class, with a low clay content in a narrow range of 1–3 % and a silt fraction in the range of 6–8 %, both in the soil layers 0–10 and 0–30 cm (Table 2). Arable soils in the surroundings of the tailings pond have a reaction of the surface layer close to neutral (site 0) due to regular liming. After the afforestation plowing, near-neutral pH is maintained only in the soil of the ridges (pH range 6.5–6.6, regardless of the depth of sampling), whereas the soil reaction in the furrows is slightly more acidic, especially in the samples from a depth of 0–30 cm. The pH value increases with time, both in soil of ridges (to pH 7.3) and furrows (to pH 6.7). Such pH growth is not surprising in the furrows as it can be explained by the transfer (erosion and accumulation) of the less acidic soil from the ridges. However, the increase of soil pH in ridges can be explained only by the permanent inflow of an alkaline dust from the landfill because the forest soil in Poland is not limed and pine stands have an acidifying influence on soil (Medynska and Kabala 2010). Organic carbon content was significantly higher in soil on ridges than in furrows, both in samples collected at a depth of 0–10 and 0–30 cm (Table 2). The carbon content increased with time: while the soil at site 1 (4 years after plowing and pine planting) contained 0.42–0.59 % of organic carbon (in layers 0–10 and 0–30 cm, respectively), the soil at site 3 (15 years after plowing and tree planting) contained 0.91–0.94 % of organic carbon (respectively, in layers 0–10 and 0–30 cm). Initially, the organic matter content in the layer 0–30 cm was significantly higher than in layer 0–10 cm layer, but with time this difference disappeared and 15 years after the plow was statistically insignificant. Differences between layers 0–10 and 0–30 cm are the result of topsoil reversal by deep plowing. The lower part of the “original” humus layer, generally poorer in organic matter, was turned and plowed onto the surface, whereas the upper part of “original” humus layer (richer in organic matter) was “trapped” within the inner zone of the newly formed doubled humus layer—apparently the ridge. It must be stressed that all presently afforested sites were abandoned before the tree planting. Natural succession of grasses and other herbs during the few years of an “abandonment period” may result in an additional enrichment in the organic matter of the superficial soil layer. Stripe plowing of such humus horizon leads to an atypical accumulation of its enriched part on contact with the unplowed and reversed plowed layer. In the furrows, conversely, more organic carbon was found in soil layer 0–10 cm than in the 0–30 cm layer, but only at site 1 was the difference statistically significant. During the 15-year period after plowing, the difference between the carbon content in soil of ridges and furrows also significantly decreased. Under the 4-year-old stand, the ridge soil contained at least twofold more carbon than that in a furrow (in layer 0–30 cm), under the 8-year-old stand, carbon content in the ridge was 44 % higher than in the furrow, while under the 15-year-old stand, only about 23 %. This confirmed that 15 years after the plow and tree planting, there is still a significant difference in organic carbon content in the soil of furrows and ridges. This difference persisted despite larger accumulation of forest litter in the furrows, and a doubling of organic carbon content in the soil of the furrow.

**Table 2:** Particle size distribution, pH, and the content of organic carbon in soils under study

Site	Soil layer	Particle size fraction (mm)			Texture class	Ridges		Furrows	
		Sand	Silt	Clay		TOC	pH	TOC	pH
		2.0–0.05	0.05–0.002	<0.002					
	cm	%				%		%	
0	0–10	90.5	6.4	3.1	S	0.58a	6.8	–	–
	0–30	91.4	6.2	2.4	S	0.43b	6.7	–	–
1	0–10	88.5	8.3	3.2	S	0.42a^a^	6.5	0.31A^b^	6.4
	0–30	88.5	8.4	3.1	S	0.59b^a^	6.6	0.26B^b^	6.1
2	0–10	92.5	6.1	1.4	S	0.71c^a^	6.7	0.50C^b^	6.2
	0–30	92.5	6.2	1.3	S	0.65c^a^	6.6	0.45C^b^	5.7
3	0–10	91.7	7.2	1.1	S	0.94d^a^	6.8	0.78D^b^	6.7
	0–30	91.6	7.3	1.1	S	0.91d^a^	7.3	0.74D^b^	6.6

Site “0” has a flat microrelief, not differentiated into ridges and furrows. Number of samples used for calculations was *N* = 15 in each layer. Homogeneous groups of mean values calculated by Duncan’s multiple range test (significant at *p* < 0.05): lowercase letters represent a comparison of the layers 0–10 and 0–30 cm in the ridges of the same age, uppercase letters represent comparison of the layers 0–10 and 0–30 cm in the furrows of the same age. ^a,b^Comparison of the same layers (0–10 or 0–30 cm) in ridges and furrows. The same letters indicate no significant difference between the means (a “homogenous group”)

Spatial and temporal differentiation of copper content in the soils at the study sites was very similar to the distribution of organic carbon. First, there was a statistically significant difference in the copper content in the soils of ridges and furrows at all three locations and in both compared soil layers (Table 3). This difference in the 0–30-cm layer at site 1 was exactly threefold (31.5 versus 10.5 mg kg^−1^ in the soil of the ridge and furrow, respectively) and clearly decreased with time; however, at site 3 (15 years after the afforestation plowing) there was still at least 60 % more copper in the soil of ridges than in furrows.

**Table 3:** Variability of the total copper concentration in soils of the ridges and furrows (*N* = 15 samples in each layer)

Site	Soil layer	Ridges				Furrows				Average content
		Mean	Min–max	SD	*v*	Mean	Min–max	SD	*v*	
	cm	mg kg^−1^			%	mg kg^−1^			%	mg kg^−1^
0	0–10	–	–	–	–	–	–	–	–	29.1
	0–30	–	–	–	–	–	–	–	–	26.5
1	0–10	27.9a^a^	25.5–32.5	2.61	9.5^d^	18.5B^b^	9.5–24.0	5.62	30^e^	23.2
	0–30	31.5a^a^	29.5–34.0	1.52	4.8^c^	10.5A^b^	6.0–12.5	2.37	22^e^	21.0
2	0–10	43.5b^a^	34.0–58.0	8.60	20^e^	30.2C^b^	26.5–32.0	2.17	7.2^c^	36.9
	0–30	39.3b^a^	33.0–43.5	3.60	9.2^d^	15.8B^b^	13.0–21.5	3.36	21^e^	27.6
3	0–10	47.5c^a^	40.5–59.0	7.28	15^d,e^	30.8C^b^	18.5–40.0	7.77	25^e^	39.2
	0–30	37.0b^a^	34.5–41.5	2.72	7.4^c^	21.5B^b^	16.0–31.0	5.37	25^e^	29.3

*SD* standard deviation, *v* coefficient of variation (*v* = SD/mean)

Homogeneous groups of mean values calculated by Duncan’s multiple range test (significant at *p* < 0.05): lowercase letters represent comparison of the layers 0–10 and 0–30 cm in the ridges of the same age, uppercase letters represent comparison of the layers 0–10 and 0–30 cm in the furrows of the same age. ^a,b^Comparison of the same layers (0–10 or 0–30 cm) in ridges and furrows. ^c,d,e^Comparison of the *v* coefficients. The same case letters indicate no significant difference between the means (a “homogenous group”)

The depth of soil sampling for analysis was important only in the furrows, where across all sites (4-, 8-, and 15-year-old stands) significantly more copper was reported in the 0–10-cm layer than in layer 0–30 cm (Table 3). This difference in copper content in layers 0–10 and 0–30 cm (in the furrows) decreased with time from an initial 180 to 150 %. In the ridges, conversely, a higher concentration of copper was initially found in layer 0–30 cm, but under the 8-year-old forest, the proportions changed and a higher copper content was present in layer 0–10 cm. This difference grew with time and at site 3 (under the 15-year-old stand) reached almost 25 % and was statistically significant.

**Fig. 3:**
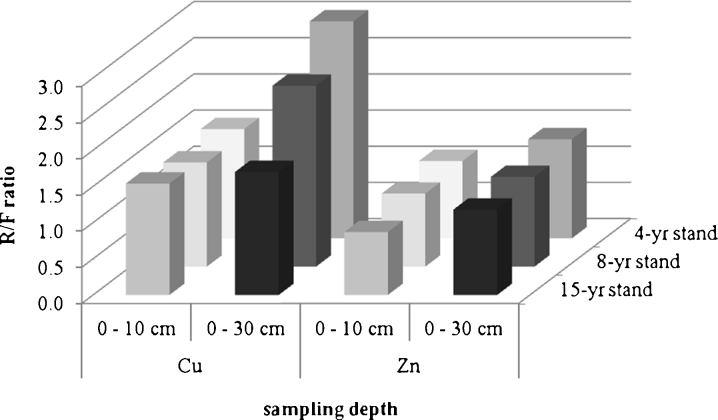
Temporal changes in the ratio of Cu and Zn content in soils of ridges and furrows. *R/F ratio* the ratio of metal content in the soil of a ridge to its content in the soil of a furrow

The third observed rule relates to an increase in the copper content over time that was observed in almost all the analyzed layers, except the 0–30-cm layer in the ridges (Fig. 3). The copper concentration, both in the ridges and furrows, increased in the period 4–15 years after the plow by about 70 % in soil layers 0–10 cm and up to 205 % in soil layers 0–30 cm in the furrows.

The average copper content calculated using all samples taken in an equal quantity from ridges and furrows (in total 30 samples at each site at two depths) confirms the above-described patterns: (1) the metal content is higher in samples collected at the 0–10-cm depth than 0–30 cm, and this difference increases with time, (2) the total content of copper in the afforested soils increases with time, both in the 0–10- and the 0–30-cm layer, clearly indicating the increase in soil contamination with this element, and (3) the rate (dynamics) of contamination increase is higher in the 0–10-cm than in the 0–30-cm soil layer.

The copper content found in soil layer 0–30 cm under the 15-year-old stand accounted for one third of the permitted copper content (that is 100 mg kg^−1^) and despite the documented elevated concentration is not contaminated with heavy metals according to the Polish legal regulations (Dz.U.2002.165.1359) and does not require reclamation. An increase in the copper content in the afforested soils could be explained by the natural bioaccumulation of the metal in the pine needles, then in forest litters, and finally in the surface mineral soil layer (Bergkvist et al. 1989), but in the studied area, it is primarily the result of contamination by the dust permanently emitted from the tailings pond and transported by the winds. Copper concentration in the falling biomass and in forest litters is strictly dependent on the distance from the tailings pond and on the height of the trees, which affects their ability to filter the dust from the air (Medynska and Kabala 2010).

Spatial variation of the copper content, expressed as the coefficient of variation (*v* = SD/mean), was significantly higher in the soil of furrows than of ridges at each study site, and generally was larger in layer 0–10 cm than 0–30 cm. Paradoxically, the lowest (in layer 0–30 cm on the ridges) and the highest (in layer 0–10 cm in the furrows) variation coefficients were found at site 1—4.8 and 30 %, respectively.

The total concentration of zinc in the soils at all the compared sites remained at levels similar to the copper content, which signifies a much lower contamination with this metal (in relation to limit values). It was also found that the influence of an afforestation plowing on the zinc distribution in topsoil is generally less significant than it is in the case of copper. Although the zinc content was generally higher in soil of the ridges than furrows, which has its justification in the natural bioaccumulation of metals in the soil humus layer, the differences were statistically significant only in the samples collected at the depth 0–30 cm (Table 4). The largest difference reaching 40 % was found at site 1 (at the concentrations 35.3 and 25.6 mg kg^−1^, in the soil of ridge and furrow, respectively), that was 4 years after afforestation plowing. Over time, this difference disappeared, and after 10 years, zinc content in the soil of furrows may even exceed its content in the soil of ridges (in the layer 0–10 cm). In soil layer 0–30 cm, the relative difference between ridges and furrows can stabilize after a few years at a level of 20 % (that also means a permanently higher Zn content in the soils of ridges than furrows).

**Table 4:** Variability of the total zinc concentration in soils of the ridges and furrows (*N* = 15 samples in each layer)

Site	Soil layer	Ridges				Furrows				Average content
		Mean	Min–max	SD	*v*	Mean	Min–max	SD	*v*	
	cm	mg kg^−1^			%	mg kg^−1^			%	mg kg^−1^
0	0–10	–	–	–	–	–	–	–	–	31.4
	0–30	–	–	–	–	–	–	–	–	25.5
1	0–10	34.1a^a^	31.5–38.0	3.09	9.1^d^	31.7A^a^	25.5–40.0	5.02	16^e^	32.9
	0–30	35.3a^a^	32.5–37.5	1.88	5.3^c^	25.6A^b^	20.5–30.5	3.41	13^d,e^	30.5
2	0–10	24.3b^a^	20.0–28.5	3.11	13^d,e^	23.9A^a^	20.5–30.5	3.63	15^e^	24.1
	0–30	26.3b^a^	23.5–30.5	2.50	9.5^d^	21.2A^a^	19.0–26.0	3.39	16^e^	23.8
3	0–10	21.6b^a^	16.0–26.5	4.71	22^e^	24.8A^a^	16.0–31.5	6.01	24^e^	23.2
	0–30	26.0b^a^	24.5–27.5	1.10	4.2^c^	21.9A^b^	19.0–24.5	1.93	8.8^d^	24.0

Explanations as in Table 3

The depth of the soil sampling for analysis (0–10 versus 0–30 cm) had no significant influence on the determination of zinc content at the site (Table 4). Zinc content in the soils of furrows was slightly higher in soil layer 0–10 cm than 0–30 cm, but in contrast to copper, these differences were not statistically significant and did not change over time. In the soils of ridges, also in contrast to copper, the zinc content was slightly higher in layer 0–30 cm, and this difference grew with time due to a decrease in zinc content in superficial soil layer 0–10 cm (in the ridges). At the same time, any changes in zinc content in the soils of furrows were measurable only during the first decade after the plow and pine planting, and in subsequent years, the zinc amounts were relatively stable, both in layer 0–10 and 0–30 cm. As a result, the mean zinc concentration, calculated using all the samples collected in equal quantities in the ridges and furrows, was almost the same at the two compared sampling depths (layers 0–10 and 0–30 cm). Moreover, the mean zinc content at the study sites, in contrast to the copper content, decreased during the first 4–8 years after afforestation plowing, and then stabilized at a (relatively) low level of 23–24 mg kg^−1^. Such a zinc level is less than one tenth of the value permissible by the Polish standard of soil quality (act No Dz.U.2002.165.1359) and generally is considered to be natural in sandy soils (Kabata-Pendias 2011).

Spatial variability of zinc content, expressed as the variation coefficient (*v* = SD/mean), was as in the case of copper higher in soils of furrows than of ridges, and was generally higher in layer 0–10 cm than 0–30 cm. It was also found that there is no clear time trend in layer 0–30 cm, whereas in layer 0–10 cm, the coefficient of variation of the zinc content significantly increases with time. The observed distribution of zinc confirmed the natural origin of this element and the little or zero influence of the tailings pond on its concentration in soils. Obviously, a certain amount of this element is detected in the dust falling on the pine needles, in the biological fall (collected at the soil surface), and in the forest litter (Medynska and Kabala 2010), but it is probably too low a concentration to stabilize in the surface layers, and is totally involved in the natural circulation and partly leached into the deeper soil layers.

## Conclusions

Results from these studies conducted over a 15-year-long chronosequence at afforested post-arable sites exposed to the impact of a large tailings pond collecting the wastes from a copper ore enrichment plant lead to the following conclusions:

1. Wide-furrow plow made before afforestation contributes to the regular spatial variation (strip-type) in soil properties, including organic carbon content and soil pH. Both the specific microrelief and spatial variability of soil properties are gradually blurred, but do not disappear over a period of at least 15 years.
2. Differences in copper content in the soil of ridges and furrows created by afforestation plowing initially reached 300 %, and after 15 years dropped significantly, but not below 60 %.
3. Estimated time of decline in the furrow-and-ridge microrelief and the differences in topsoil properties cover at least 30–40 years after afforestation plowing.
4. “Wide-furrow plow” has little impact on the variability of zinc concentration which is not influenced by the emissions from the tailings pond and was found to be close to natural in the soils under study.
5. Soil sampling in the contaminated sites with “furrow-and-ridge” microrelief must include the collection of an appropriate number of soil samples in equal quantities both from furrows and ridges to allow a reliable estimation of mean trace element concentrations and time-dependent trends.
